# Melanoma incidence and mortality in Scotland 1979–2003

**DOI:** 10.1038/sj.bjc.6603801

**Published:** 2007-05-29

**Authors:** R M MacKie, C Bray, J Vestey, V Doherty, A Evans, D Thomson, M Nicolson, for and on behalf of the Scottish Melanoma Group

**Affiliations:** 1 Department of Public Health and Health Policy, University of Glasgow, Glasgow G12 8RZ, UK; 2 NHS Greater Glasgow and Clyde, Dalian House, Glasgow G3 8YZ, UK; 3 Department of Dermatology, Raigmore Hospital, Inverness IV2 3UJ, UK; 4 Department of Dermatology, Lauriston Building Royal Infirmary of Edinburgh, Edinburgh EH3 9HA, UK; 5 Department of Pathology, Ninewells Hospital, Dundee DD1 9SY, UK; 6 Department of Pathology, Aberdeen Royal Infirmary, Aberdeen AB25 2ZN, UK; 7 Department of Oncology, Aberdeen Royal Infirmary, Aberdeen AB25 2ZN, UK

**Keywords:** melanoma, incidence, mortality, survival, Scotland

## Abstract

We studied 12 450 cases of invasive melanoma diagnosed in Scotland in 1979–2003, by thickness, pathological type, and body site at ages under 40, 40–59, and 60 years and over. Melanoma incidence trebled in males from 3.57 to 10.93/10^5^ per year, and increased 2.3-fold in females from 5.60 to 12.96/10^5^ per year. The rate of increase fell in each successive 5-year period. The greatest increase was in males aged 60 years and over at diagnosis. Significant incidence increases were seen in melanomas <1 mm in all three age groups, but those >4 mm only increased significantly at ages 60 years and over. All histological types increased significantly at ages 60 years and over, and in this age group the greatest increase was seen on the head and neck. Five-year disease-free survival improved steadily. Survival figures for 1994–1998 ranged from 93.6% for males and 95.8% for females with tumours <1 mm, to 52.4 and 48.3%, respectively, for those with tumours >4 mm. Over the 25 years, melanoma mortality doubled in males from 1.1 to 2.4/10^5^ per year, but was unchanged in females at 1.5/10^5^ per year. Public education on melanoma is required both for primary prevention and earlier diagnosis, particularly for older males.

Cutaneous melanoma is a cause of concern for those involved in cancer control in Europe (de Vries *et al*, 2003; Hemminki *et al*, 2003; de Vries and Coebergh, 2004), North America (Jemal *et al*, 2001; Swetter *et al*, 2005), and Australasia (Martin and Robinson, 2004; Baade and Coory, 2005; Coory *et al*, 2006). Data from all three continents show a continuing rise in incidence, although in some countries incidence figures may be stabilising in younger females. Mortality shows a slower rise than incidence and even a falling trend in some countries (Bosetti *et al*, 2004; Schaffer *et al*, 2005). Since 1979, The Scottish Melanoma Group has kept detailed population-based records of all new cases of invasive melanoma diagnosed in Scotland in both the National Health Service and the private sector. These data differ from that gathered by the Scottish Cancer Registry by inclusion of tumour thickness. At a time when there is concern over the rising incidence of melanoma, termed ‘the melanoma epidemic’ in the US (Schaffer *et al*, 2005), change in the incidence by tumour thickness is a useful indicator of probable future morbidity and mortality resulting from patients with thicker tumours who are likely to progress to AJCC stage 3 and 4 disease.

In this paper, we present a 25-year report of the changing melanoma incidence and mortality in Scotland to complement previous reports (Mackie *et al*, 1985, 1992, 1997, 2002), together with relative changes in incidence by sex, age group, tumour thickness, and histological type. Such data may guide future public health messages aimed at prevention and earlier diagnosis.

## METHODS

Pathologists in Scotland (latitude 55–59° North, population 5.1 million) supply information on all newly diagnosed invasive cutaneous melanomas (Clark level 2 or deeper), including tumour thickness, histological type, body site, and other details. Cases recorded in this way by the Scottish Melanoma Group are cross-checked with the Scottish Cancer Registry to ensure completeness of registration. All cases pathologically ratified by both sources are included in the analysis. Information on treatment and follow-up is obtained from the relevant clinician at regular intervals. Causes of death are checked with the death certificates, and rarely, when cases are lost to follow-up.

Cases were considered in three age groups: under 40, 40–59, and 60 years and over at diagnosis, by histological type as superficial spreading melanoma, nodular melanoma, lentigo maligna melanoma, and acral melanoma. Finally, four main body sites of head and neck, trunk, upper limb, and lower limb were considered.

To ensure an adequate number of cases for analysis when further subdivided by tumour thickness, histological type, and body site, we have reported results in 5-year periods: 1979–1983, 1984–1988, 1989–1993, 1994–1998, and 1999–2003. Incidence rates for the whole group were calculated and also for the three age groupings at diagnosis, all age-standardised to the European population (EASR). Changes in overall incidence rates between the first and last quinquennia were calculated for males and females, and by tumour thickness, pathological subtype, and body site. The number of cases was assumed to follow a Poisson distribution and the *P*-value for the ratio of the two rates was based on a log-linear model using Wald's method (Price and Bonnet, 2000). Mean annual percentage changes in incidence within each 5-year period were calculated and compared with each successive cohort for all patients and by age group.

The association between 5-year survival and each 5-year period was assessed using *χ*^2^ tests for trend.

## RESULTS

Table 1 gives details for the five quinquennia, with a total of 12 450 cases, 4810 males and 7640 females. Between 1979 and 2003, incidence trebled in males from 3.57 to 10.93/10^5^ per year, and rose 2.3-fold in females from 5.6 to 12.96/10^5^ per year (each *P*<0.001). The female:male ratio is 1.6 : 1 for the whole time period, falling from 2.1 : 1 in 1979 to 1.3 : 1 in 2003. The annual percentage increase in incidence declined from a mean of 11.3% for both sexes in the first 5-year period to a mean of 2.95 and 2.1% for males and females, respectively, in 1999–2003.

Table 2 divides the cases further by quinquennium, by sex, and into three age groups at diagnosis under 40, 40–59, and 60 years and over. Over the 25-year period, a steady upward trend in incidence is seen in both sexes and all age groups, which is most marked in those aged 60 years and over where in 1998–2003 the male incidence at 34.17 exceeds that of the females at 29.62/10^5^ per year. The mean annual percentage change in incidence in each 5-year group by age and sex shows that in all three age groups. The steepest rise in incidence and least reduction in rate of rise was seen in males aged 60 years and over.

While the number of cases with AJCC stage 3 or 4 at diagnosis increased over the 25 years, they comprise only 255 (2%) of cases, with the majority aged 60 years and over at diagnosis.

**Figure 1:**
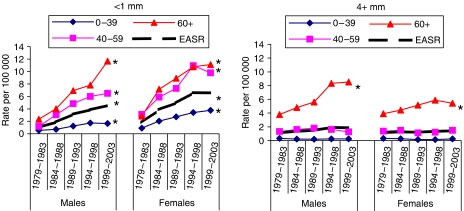
Changes in melanoma incidence 1979–2003 by age group for primary tumours <1.0 and 4 mm and over at diagnosis. ^*^P<0.001 when 1979–1983 and 1999–2003 cohorts are compared.

Table 3 shows incidence in each 5-year period by thickness, <1.0, 1–1.99, 2–2.99, 3–3.99, and 4 mm and over, and Figure 1 shows the incidence changes by age for the two groups under 1 and 4 mm and over. Overall, 43% of cases were less than 1 mm at diagnosis, but the proportion in this good prognosis category has increased significantly over time. In the first quinquennium (1979–1983), 28% of melanomas in males and 27% in females were under 1 mm, while in the 1999–2003 quinquennium, 43% of males and 50% of females had tumours under 1 mm, a significant rise in both (each: *P*<0.001).

In 1979–83, 32% of males and 24% of females had melanomas 4 mm or thicker. By 1999–2003, the percentage >4 mm had fallen to 19% of males and 14% of females, a significant fall in both (*P*<0.001). However, the actual incidence of melanomas 4 mm or thicker continues to increase in females but may have stabilised in males when the most recent two quinquennia are compared. Figure 1 shows that in the 4 mm and thicker group, there are significant increases in both sexes only in those aged 60 years and over.

The greatest increase is seen in superficial spreading melanomas in both sexes and all age groups (Table 4). This tumour type comprises 59% of all cases and is the only type to have increased significantly in all three age groups over the 25 years (*P*<0.001). Lentigo maligna melanomas comprise 11% of all cases, and have increased significantly in males aged 40–59 and 60 years and over, and in females aged 60 years and over (*P*<0.001, for all). Nodular melanomas comprise 20% of cases in males, and 16% in females, and have increased significantly only in the over 60s of both sexes (each: *P*<0.001). Acral melanomas comprise 10.3% of all melanomas in males and 9.5% in females, and have increased significantly in both sexes in the under 40 and 60 years and over age groups (*P*<0.001 for all). (Data not shown)

**Figure 2:**
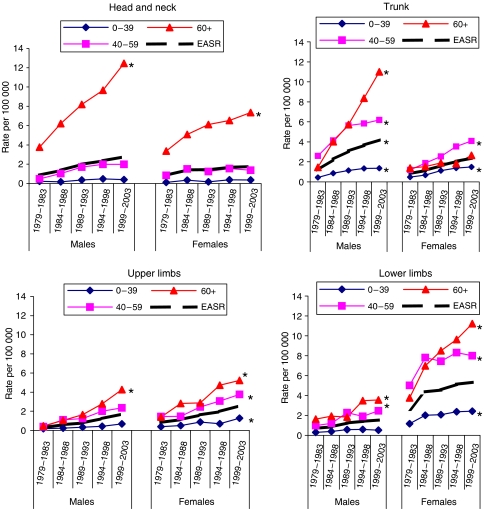
Changes in melanoma incidence 1979–2003 by body site and age group. ^*^P<0.001 when 1979–83 and 1999–2003 cohorts are compared.

The trunk continues to be the commonest primary site for males and the lower limb for females (Table 5), but Figure 2 shows that there has been a steep rise in head and neck lesions in those aged 60 years and over in both sexes (*P*<0.001). The greatest increase has been in upper limb lesions, from 0.28 to 1.70/10^5^ per year for males and 0.83 to 2.58/10^5^ per year for females.

### Survival and mortality

**Figure 3:**
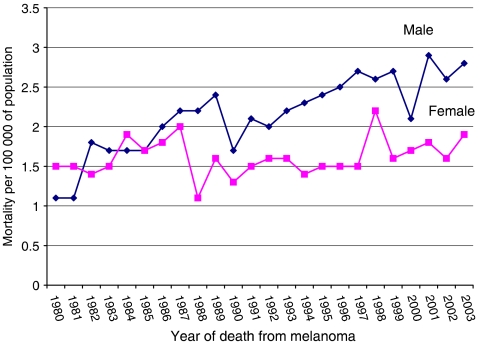
Mortality from melanoma of the skin in Scotland 1980–2003.

Survival from melanoma within 5 years of diagnosis is shown in Table 6 by gender and primary tumour thickness in 5-year periods. There were 1066 patients who died of non-melanoma causes, and these have been censored. Only 189 patients (2%) were lost to follow-up, usually because they left the UK. As expected, survival rates are highest in the thinnest tumour group and fall with each 1 mm increase in tumour thickness. It is notable first that within each thickness grouping, there has been a continuous and significant improvement in survival (*P* for trend=0.001 for males, 0.005 for females). Five-year survival rates for 1994–1998 range from 93.6 and 95.8%, respectively, for males and females with tumours under 1 mm at diagnosis, to 52.4 and 48.35% for males and females with tumours 4 mm or thicker at diagnosis. In all thickness groups up to 4 mm, females have significantly superior survival to males (*P*<0.001). Figure 3 shows mortality, which has doubled in males from 1.1/10^5^ to 2.4/10^5^ per year and remained constant at 1.5 10^−5^ per year for females.

## DISCUSSION

We show that between 1979 and 2003 the incidence of invasive cutaneous melanoma in Scotland trebled in males and more than doubled in females, the rise being most marked in those aged 60 years and over at diagnosis. In this age group, incidence in Scotland is now greater in males than in females, a pattern not reported before in the UK.

The general pattern worldwide is that in relatively low-incidence countries female exceeds male incidence, but in higher-incidence countries such as Australia, the incidence is either equal, or there is a male preponderance (Coory *et al*, 2006). Thus, Scotland appears to be moving toward the pattern of a high-incidence country. A similar reversal has also been observed in New Zealand (Martin and Robinson, 2004), but at a higher baseline level and may suggest that females are more disposed to practice sensible patterns of sun exposure with a consequently slower rate of increase in older females than males. If this continues and is seen in other countries, it adds to the evidence that recent sun exposure contributes to melanoma risk as well as the established importance of childhood and early adulthood exposure. This is of obvious importance as it strengthens the case for sun protection messages directed at older sections of the population.

The annual percentage change in our Scottish data shows that the rate of incidence increase is levelling out, falling from 11.2% per year for males and 11.3% per year for females when 1979–1983 figures are compared with 1984–1988 to 2.9% per year for males and 2.1% per year for females when 1994–1998 figures are compared with 1999–2003. This pattern is also seen in other Northern and Western European countries, but contrasts with that in Southern and Eastern Europe, where rates of incidence continue to rise rapidly (de Vries *et al*, 2003). The reasons for these contrasting patterns are not clear but could be due to greater knowledge of the damaging effects of sun exposure in the paler more vulnerable populations of Northern and Western Europe resulting in an altered attitude and behaviour to sun exposure.

A continuing increase in melanoma incidence has been predicted for both the UK and the Netherlands. Diffey (2004) has suggested that any downturn in UK incidence attributable to primary prevention activities may not be seen for 30 years, and de Vries *et al* (2005) predicts an 80% increase in the Netherlands by 2015, both predictions taking account of the ageing population. Our data do not contradict these predictions but the deceleration in the rate of increase suggests that there will be smaller increases than they forecasted.

### Age groups

We show in Scotland a continuing incidence increase in both sexes and all three age groups, greatest in both sexes at ages 60 years and over. In contrast to Scandinavia (de Vries *et al*, 2003), we have no evidence of a downward trend in incidence in younger females. Many in Scotland's ageing population currently retire relatively early and spend a proportion of the winter months in warmer climates, so incidence in this age group may increase still further in the future, requiring the burden of melanoma care to be increasingly directed toward older people.

### Thickness

Our data show that the overall increase in incidence is significant not only for melanomas less than 1 mm at diagnosis, seen in all three age groups, but also for thick tumours in those aged 60 years and over. It has been suggested that much of the reported worldwide increase in incidence reflects inappropriate pathological interpretation of melanocytic lesions under 1 mm thick as melanoma rather than a benign melanocytic proliferation, which include ‘lesions which lack the potential for metastasis’ (Burton and Armstrong, 1998). The fact that in Scotland the increases affect all thickness categories argues that the rise in incidence of potentially fatal melanoma is real and not an artefact.

### Histopathological subtype

The only subtype that has increased significantly in all age groups over the 25 years is superficial spreading melanoma, and again here the steepest increase is in males and females aged 60 years and over at diagnosis. Over the 25-year period, nodular melanomas have increased significantly only in males and females aged over 60 years. This should be borne in mind in early detection campaigns as nodular melanomas tend to be thicker than other subtypes, and current public education concentrates mainly on the less aggressive superficial spreading type (Demierre *et al*, 2005). Lentigo maligna melanoma has increased in those aged 60 years and over, with the steepest rise from 1994–1998 to 1999–2003. This trend suggests an effect of total cumulative lifetime UV exposure, which is believed to be of greater aetiological importance for lentigo maligna melanoma than for other histological variants.

As in other Caucasian populations, we recorded a low incidence of acral melanomas, but there were significant increases in those aged under 40 and 60 years, and over in both sexes.

### Body site

The steepest incidence increases were at age 60 years and over, involving the head and neck and upper limb in both sexes, the trunk in males, and the lower limb in females. If one assumes that the head and neck in both sexes is a constantly UV-exposed site whereas the trunk is an intermittently exposed site, then it would appear that both the intermittent intense UV exposure associated with sunny vacations, and chronic lifetime exposure are contributing to the increase. Our observation that 50% of the head and neck increase is due to lentigo maligna melanomas, and 50% to superficial spreading and nodular lesions is further evidence that this is the case.

Our site-related data suggest that while for all adults the male trunk and female lower limb continue to merit stress in future public awareness campaigns, head and neck lesions also require emphasis for those aged 60 years and over.

### Survival and mortality

Our data on 5-year survival show not only the well-recognised significant survival advantage for patients with thinner melanomas, but also improvement in survival over 20 years within each thickness category. Not all melanoma recurrences will be apparent in the first 5 years of follow-up, but the proportion recurring later is low. The reasons for the steadily improving survival after controlling for thickness are not clear. Before 1998, neither sentinel node biopsy nor adjuvant interferon therapy were standards of care in Scotland, and therefore, even if ongoing trials on these approaches eventually report a significant survival advantage, they cannot explain the pattern observed for patients diagnosed between 1979 and 1998. The female survival advantage within each thickness category up to tumours 4 mm and thicker has also been reported from North America among 1829 patients (Scoggins *et al*, 2006) and its explanation merits further study.

Melanoma mortality over the 25-year period of observation has more than doubled in males from 1.1 to 2.4/10^5^ per year, with more male than female deaths since 1985. In contrast, female mortality has remained constant at 1.5/10^5^ per year. These data indicate an improvement in case fatality given the three-fold rise in incidence in males, and 2.4-fold rise in females. This pattern of rapidly rising incidence but more slowly rising mortality suggests that a melanoma awareness campaign in Scotland in the mid 1980s had some effect in encouraging earlier surgical excision of thinner lesions (Doherty and Mackie, 1988). The static mortality in females despite the male increase supports this view, as we observed a predominance of females attending dedicated pigmented lesion clinics during and after the campaigns (Mackie and Hole, 1992). The only countries in Europe reported to show a continuing increase in melanoma mortality are Scotland, France, Poland, and Hungary (Bosetti *et al*, 2004) and the fact that only Scotland showed a static mortality among females may be in part due to the early detection public education in the 1980s.

The steady rise in melanoma incidence in Scotland over a 25-year period appears likely to continue, particularly in older males. The failure of early detection activities to reach the older male is also of concern in Australia (Janda *et al*, 2006), Canada (Rivers and Gallagher 1995), and the US (Geller *et al*, 2002).

We consider that our data give a sound quantitative base for future UK-based public education planned by CRUK and others. The data indicate that primary prevention messages require to be aimed at the whole population and early detection messages should selectively target older males.

## Figures and Tables

**Table 1:** Distribution of 12 450 cases by gender and quinquennium and incidence in Scotland of melanoma age-standardised to the European population (EASR)

	**Males *n*=4810**			**Females *n*=7640**		
**Year**	**Cases**	**Incidence**	**APC**	**Cases**	**Incidence**	**APC**
1979–1983	447	3.57		906	5.60	
1984–1988	712	5.59	11.3	1312	8.75	11.2
1989–1993	954	7.75	7.7	1520	9.94	8.2
1994–1998	1217	9.53	4.5	1829	11.89	3.9
1999–2003	1480	10.93	2.9	2073	12.96	2.1

APC=Mean annual percentage change in incidence by quinquennium.

**Table 2:** Number of cases (No.) and incidence in brackets adjusted to EASR of invasive melanoma in Scotland 1979–2003 by quinquennia and gender at ages under 40, 40–59, and 60 years and over at diagnosis

	**<40**		**40–59**		**60+**	
**Age group (years)**	**No.**	**APC**	**No.**	**APC**	**No.**	**APC**
*Males*						
1979–1983	87 (1.22)		144 (5.07)		181 (9.38)	
1984–1988	131 (1.73)	*8.3*	232 (8.19)	*12.3*	304 (14.96)	*11.9*
1989–1993	189 (2.50)	*8.9*	335 (11.44)	*7.9*	420 (20.26)	*7.1*
1994–1998	226 (3.00)	*4.0*	390 (12.64)	*2.1*	596 (27.53)	*7.2*
1999–2003	222 (3.09)	*0.6*	458 (13.72)	*1.6*	783 (34.17)	*4.8*
						
*Females*						
1979–1983	160 (2.24)		279 (9.08)		352 (11.70)	
1984–1988	273 (3.65)	*10.8*	405 (13.60)	*10.0*	570 (18.73)	*12.0*
1989–1993	333 (4.34)	*3.8*	440 (14.29)	*1.0*	726 (22.56)	*4.1*
1994–1998	386 (4.94)	*2.8*	572 (17.82)	*4.9*	868 (26.67)	*3.6*
1999–2003	432 (5.80)	*3.5*	629 (18.19)	*0.4*	978 (29.62)	*2.2*

Mean annual percentage change (APC) in italics. Year of birth for 349 (2.8%) subjects not available.

**Table 3:** Melanoma in Scotland: numbers and incidence (brackets) adjusted to EASR by quinquennia and tumour thickness

**Thickness**	**<1 mm**	**1–1.99 mm**	**2–2.99 mm**	**3–3.99 mm**	**4+ mm**
*Males*					
1979–1983	125 (1.01)	64 (0.53)	53 (0.43)	55 (0.42)	142 (1.14)
1984–1988	231 (1.88)	147 (1.14)	80 (0.66)	63 (0.51)	178 (1.34)
1989–1993	383 (3.13)	192 (1.57)	102 (0.84)	80 (0.63)	186 (1.50)
1994–1998	484 (3.85)	237 (1.85)	127 (1.00)	82 (0.62)	245 (1.91)
1999–2003	607 (4.56)	307 (2.30)	139 (1.03)	90 (0.64)	260 (1.86)
					
*Females*					
1979–1983	270 (1.81)	179 (1.18)	133 (0.83)	101 (0.58)	217 (1.17)
1984–1988	553 (3.89)	289 (2.03)	129 (0.91)	96 (0.60)	235 (1.27)
1989–1993	715 (4.94)	316 (2.18)	148 (0.98)	98 (0.58)	234 (1.22)
1994–1998	938 (6.61)	341 (2.28)	165 (0.97)	87 (0.48)	264 (1.35)
1999–2003	984 (6.58)	456 (2.95)	165 (1.00)	91 (0.49)	284 (2.42)

Thickness data missing for 48 subjects and not relevant for 255 who presented with stage 3 or 4 disease.

**Table 4:** Melanoma in Scotland 1979–2003: numbers and incidence (brackets) adjusted to EASR by quinquennia and histogenetic type

**Type**	**SSM**	**LMM**	**Nodular**	**Acral**
*Males*				
1979–1983	200 (1.64)	54 (0.44)	117 (0.90)	62 (0.50)
1984–1988	364 (2.93)	80 (0.60)	169 (1.31)	73 (0.56)
1989–1993	515 (4.22)	115 (0.89)	189 (1.58)	101 (0.78)
1994–1998	677 (5.37)	129 (0.92)	243 (1.90)	112 (0.86)
1999–2003	851 (6.46)	187 (0.94)	228 (1.62)	148 (1.06)
				
*Females*				
1979–1983	449 (3.08)	126 (0.62)	209 (1.18)	106 (0.62)
1984–1988	758 (5.54)	155 (1.77)	256 (1.59)	121 (0.70)
1989–1993	927 (6.61)	182 (0.91)	240 (1.44)	146 (0.85)
1994–1998	1198 (8.37)	168 (0.81)	257 (1.51)	156 (0.90)
1999–2003	1304 (8.82)	231 (1.12)	269 (1.49)	201 (1.09)

LMM=lentigo maligna melanoma; SSM=superficial spreading melanoma.

377 subjects (3%) not included as histogenetic type either not classifiable or belonging to a rare group, for example, mucosal.

**Table 5:** Melanoma in Scotland: numbers and incidence (brackets) adjusted to EASR by quinquennia and body site

**Body site**	**Head and neck**	**Trunk**	**Upper limbs**	**Lower limbs**
*Males*				
1979–1983	118 (0.87)	144 (1.19)	37 (0.28)	82 (0.70)
1984–1988	176 (1.36)	280 (2.25)	72 (0.59)	113 (0.86)
1989–1993	247 (1.98)	375 (3.08)	97 (0.78)	150 (1.24)
1994–1998	306 (2.35)	464 (3.67)	155 (1.23)	180 (1.41)
1999–2003	385 (2.77)	559 (4.20)	224 (1.70)	199 (1.53)
				
*Females*				
1979–1983	173 (0.83)	128 (0.86)	138 (0.83)	379 (2.62)
1984–1988	259 (1.42)	155 (1.17)	167 (1.13)	608 (4.38)
1989–1993	276 (1.41)	226 (1.69)	231 (1.62)	655 (4.55)
1994–1998	318 (1.68)	278 (2.06)	293 (1.97)	750 (5.13)
1999–2003	356 (1.76)	338 (2.42)	385 (2.58)	796 (5.34)

1178 (9%) evenly distributed between quinquennia and sex excluded as on less common sites such as mucosal surfaces.

**Table 6:** Percentage 5-year melanoma-free survival for 8897 patients diagnosed 1979–1998 by gender and tumour thickness

**Thickness**	**<1.0 mm**	**1–1.99 mm**	**2–2.99 mm**	**3–3.99 mm**	**4 mm+**
*Males*					
1979–83	73.2	68.4	55.0	40.0	33.9
1984–88	82.7	78.3	56.5	45.1	30.8
1989–93	90.2	80.0	61.4	56.1	36.2
1994–98	93.6	87.9	71.3	65.3	52.4
					
*Females*					
1979–83	86.0	84.9	62.9	55.7	37.6
1984–88	93.6	87.8	79.4	64.1	43.0
1989–93	93.5	94.9	77.4	66.2	44.8
1994–98	95.8	94.3	86.6	71.4	48.3
